# Analytic Expression of Quantum Discords in Werner States under LQCC

**DOI:** 10.3390/e22020147

**Published:** 2020-01-26

**Authors:** Chuanmei Xie, Zhanjun Zhang, Jianlan Chen, Xiaofeng Yin

**Affiliations:** 1 School of Physics & Material Science, Anhui University, Hefei 230039, China; cmxie@ahu.edu.cn (C.X.); jianlanchen@ahu.edu.cn (J.C.); xiaofengyin@ahu.edu.cn (X.Y.); 2 School of Information and Electronic Engineering, Zhejiang Gongshang University, Hangzhou 310018, China

**Keywords:** local quantum operations and classical communication (LQCC), Werner state under LQCC, quantum discord, analytical expression of quantum discord, 03.65.Ta, 03.67.-a

## Abstract

In this paper, quantum discords in a special kind of states, i.e., Werner states by local quantum operations and classical communication (LQCC) protocols (WLQCC states), are studied. Nineteen parameters to quantify the quantum discords are reduced to four parameters in terms of properties of Werner states and quantum discord. In the case of orthogonal projective measures, analytic expression of quantum discords in WLQCC states is analytically worked out. Some properties of the quantum discord in the WLQCC states are obtained, especially the variation relations between the quantum discords and the parameters characterizing the WLQCC states. By virtue of numerical computations, quantum discords in a Werner state before and after LQCC protocols are compared. It is found that quantum discord in any WLQCC state cannot exceed that in the original Werner state.

## 1. Introduction

In quantum information processing, particularly in the theoretical and experimental investigation of quantum correlations, the local quantum operations, and classical communication (LQCC) protocols play an essential role. The importance of LQCC in quantum information processing has long been recognized, playing a key role in teleportation [1], entanglement distillation [2,3], one-way [4] and distributed [5] quantum computing, local cloning [6], quantum secret sharing [7,8], and beyond. While many important results have been obtained concerning LQCC [9,10,11,12,13,14,15,16,17], it has, nonetheless, proven difficult to characterize in simple terms.

Up to now, the form of quantum correlation related to LQCC operations is focused on quantum entanglement. A distinct feature has been found that quantum entanglement cannot be increased through the LQCC channel. It is well known that quantum correlations can be classified into two forms. One form is quantum entanglement, another is quantum correlation different from quantum entanglement (QCDE). The first kind of QCDE, namely quantum discord (QD), was first revealed by Ollivier and Zurek [18] in 2001. Distinctly, it exists even in some separable states, where quantum entanglement does not emerge obviously. This distinct feature attracted many researchers further investigations. Later, other kinds of QCDEs were proposed [19,20,21,22,23,24,25,26,27,28,29,30,31] and their role in quantum information theory is standing out gradually. Moreover, the potential applications of these discovered QCDEs in quantum information processing are gradually exploited, such as quantum computation [32,33], state merging [34], remote state preparation [35], assisted state discrimination [36], quantum correlation swapping [37,38,39], etc. By far, it has been well admitted that QCDE is another form of important quantum resources. Hence, it is interesting to study the behavior of QCDEs in quantum states through LQCC operations. Particularly, similar to the case of quantum entanglement, we wonder whether LQCC can increase QCDEs in quantum states or not.

In this paper, we will study the influences of LQCC on QD in a given quantum state. Considering the difficulties in characterizing the LQCC operation and quantifying QD in a quantum state, we will originally study a comparatively simple case. To be concrete, we will study whether QDs involved in two-qubit Werner states can be increased by the single-state LQCC protocol or not. As for this specific topic, our main motivations are fourfold: (1) study the QD in the concerned states through LQCC operations quantitatively; (2) discover whether LQCC can increase QD or not similar to the quantum entanglement case; (3) if yes or no, seek out the similarities and differences among them qualitatively; (4) try to expose the quantitative features of QDs in the LQCC process. It is worth stressing that so far quantum discord in a generic state is extremely difficult to evaluate. Because of the definition of discord requires the optimization to be executed over all measurements on one subsystem. Until now, the explicit analytical results on QD are known only for a few classes of quantum states [40,41,42,43,44].

The rest of this paper is outlined as follows. In Section 2, WLQCC states are originally introduced and pointedly simplified. In Section 3, the analytical expression of QD in the WLQCC states is derived. In Section 4, some analyses and discussions about the QDs are made and some distinct features are revealed. Finally, a concise summary is given in Section 5.

## 2. WLQCC State

Usually, a two-qubit Werner state is written as [45]

(1) $$\begin{array}{c} \rho_{ab}^{W}=\frac{1-F}{3}I+\frac{4F-1}{3}|\Psi^{-}\rangle \langle \Psi^{-}|, \end{array}$$

where I denotes the 4×4 identity matrix and $|\Psi^{-}\rangle =(|01\rangle -|10\rangle )/\sqrt{2}$ is the singlet state. The Werner state $\rho_{ab}^{W}$ is characterized by a single real parameter *F* called fidelity with 1/4≤F≤. The single-state LQCC protocol maps a Werner state $\rho_{ab}^{W}$ to WLQCC state $\varrho_{ab}$ in the form of [46]

(2) $$\begin{array}{c} \varrho_{ab}=\frac{A⊗B\rho_{ab}^{W}A^{†}⊗B^{†}}{\mathrm{Tr}(A⊗B\rho_{ab}^{W}A^{†}⊗B^{†})}, \end{array}$$

where *A* and *B* are operators act on subsystems *a* and *b*, respectively. The success probability of this protocol is $\mathrm{Tr}(A⊗B\rho_{ab}^{W}A^{†}⊗B^{†})$. The only restrictions for operators *A* and *B* are $A^{†}A\leq I_{2}$ and $B^{†}B\leq I_{2}$. They can be written as $A=U_{a}f_{a}U_{a}^{\prime }$ and $B=V_{b}f_{b}V_{b}^{\prime }$, where $U_{a}$, $U_{a}^{\prime }$, $V_{b}$ and $V_{b}^{\prime }$ are arbitrary unitary operators, $f_{a}$ and $f_{b}$ are filtering operators taking the forms of $f_{a}=\sum_{i=0}^{1}\alpha_{a}^{i}\left|i\rangle \langle i\right|$ and $f_{b}=\sum_{j=0}^{1}\alpha_{b}^{j}\left|j\rangle \langle j\right|$, respectively. Considering physical realizations of filtering operators $f_{a}$ and $f_{b}$, their eigenvalues must be between zero and one, i.e., $0\leq \alpha_{a}^{i},\alpha_{b}^{j}\leq 1$. Thus any nontrivial LQCC (i.e., any LQCC which is not the zero map) for two-qubit states can be written in the form of [46]

(3) $$\begin{array}{c} A⊗B=\gamma U_{a}f_{a}^{\prime }U_{a}^{\prime }⊗V_{b}f_{b}^{\prime }V_{b}^{\prime }, \end{array}$$

where γ is a scale factor in the range 0<γ≤1, $f_{a}^{\prime }=\left(\begin{array}{cc} 1 & 0\\ 0 & \alpha_{a} \end{array}\right)$ and $f_{b}^{\prime }=\left(\begin{array}{cc} 1 & 0\\ 0 & \alpha_{b} \end{array}\right)$ with $0\leq \alpha_{a},\alpha_{b}\leq 1$. Actually, the scale factor γ can be ignored for that it will vanish in the normalizing process. In spite of the trivial parameter γ, there are still nineteen parameters required to determine a WLQCC state.

According to [47], there exists an equivalence relation $V_{b}^{\prime }\rho_{ab}^{W}V_{b}^{\prime †}=V_{a}^{T}\rho_{ab}^{W}V_{a}^{*}$ by virtue of the inherent permutation symmetry of two-qubit Werner states $\rho_{ab}^{W}$. Making use of this property, one can further reexpress the state $\varrho_{ab}$ as

(4) $$\begin{array}{c} \varrho_{ab}=\frac{U_{a}V_{b}\rho_{ab}^{k}V_{b}^{†}U_{a}^{†}}{\mathrm{Tr}(U_{a}V_{b}\rho_{ab}^{k}V_{b}^{†}U_{a}^{†})}=U_{a}V_{b}\varrho_{ab}^{k}V_{b}^{†}U_{a}^{†}, \end{array}$$

where

(5) $$\begin{array}{ccc} \varrho_{ab}^{k} & = & f_{a}^{\prime }W_{a}⊗f_{b}^{\prime }\rho_{ab}^{W}W_{a}^{†}f_{a}^{\prime }⊗f_{b}^{\prime } \end{array}$$

with $W_{a}=U_{a}^{\prime }V_{a}^{\prime T}$. Obviously, $W_{a}$ can be seen as an arbitrary unitary operator on subsystem *a* in the matrix form of $\left(\begin{array}{cc} \sqrt{\zeta }e^{i(\theta +\mu )} & \sqrt{1-\zeta }e^{i\nu }\\ -\sqrt{1-\zeta }e^{i(\theta -\nu )} & \sqrt{\zeta }e^{-i\mu } \end{array}\right)$, here ζ is in the range 0≤ζ≤1 and 0≤μ,ν,θ≤2π. After substituting the matrix forms of $\rho_{ab}^{W}$, $W_{a}$, and $W_{a}^{†}$ into Equation (5), one can achieve the matrix form of $\varrho_{ab}^{k}$ in computational bases:

(6) $$\begin{array}{cc} \varrho_{ab}^{k}\\ = & \frac{\left(4F-1\right)}{6}\left(\begin{array}{cccc} \left(1-\zeta \right)+\frac{2(1-F)}{\left(4F-1\right)} & -\alpha_{b}\sqrt{\zeta (1-\zeta )}e^{-i(\mu -\nu +\theta )} & \alpha_{a}\sqrt{\zeta (1-\zeta )}e^{i(\mu +\nu )} & \alpha_{a}\alpha_{b}\left(1-\zeta \right)e^{i(2\nu -\theta )}\\ -\alpha_{b}\sqrt{\zeta (1-\zeta )}e^{i(\mu -\nu +\theta )} & \alpha_{b}^{2}\zeta +\frac{2(1-F)}{\left(4F-1\right)}\alpha_{b}^{2} & -\alpha_{a}\alpha_{b}\zeta e^{i(2\mu +\theta )} & -\alpha_{a}\alpha_{b}^{2}\sqrt{\zeta (1-\zeta )}e^{i(\mu +\nu )}\\ \alpha_{a}\sqrt{\zeta (1-\zeta )}e^{-i(\mu +\nu )} & -\alpha_{a}\alpha_{b}\zeta e^{-i(2\mu +\theta )} & \alpha_{a}^{2}\zeta +\frac{2(1-F)}{\left(4F-1\right)}\alpha_{a}^{2} & \alpha_{a}^{2}\alpha_{b}\sqrt{\zeta (1-\zeta )}e^{-i(\mu -\nu +\theta )}\\ \alpha_{a}\alpha_{b}\left(1-\zeta \right)e^{-i(2\nu -\theta )} & -\alpha_{a}\alpha_{b}^{2}\sqrt{\zeta (1-\zeta )}e^{-i(\mu +\nu )} & \alpha_{a}^{2}\alpha_{b}\sqrt{\zeta (1-\zeta )}e^{i(\mu -\nu +\theta )} & \alpha_{a}^{2}\alpha_{b}^{2}\left(1-\zeta \right)+\frac{2(1-F)}{\left(4F-1\right)}\alpha_{a}^{2}\alpha_{b}^{2} \end{array}\right). \end{array}$$

Furthermore, after the normalizing process, one can get

(7) $$\begin{array}{c} \varrho_{ab}^{k}=\frac{\xi_{ab}}{\Omega }, \end{array}$$

where $\xi_{ab}=6\varrho_{ab}^{k}$ and

(8) $$\begin{array}{c} \Omega =\mathrm{Tr}\xi_{ab}=3\left(\alpha_{a}^{2}+\alpha_{b}^{2}\right)+\left(1-\alpha_{a}^{2}\right)\left(1-\alpha_{b}^{2}\right)\left[2\left(1-F\right)+\left(4F-1\right)\left(1-\zeta \right)\right]. \end{array}$$

Obviously, one can find that the number of parameters required to determine the state $\varrho_{ab}^{k}$ is seven, i.e., $F,\alpha_{a},\alpha_{b},\zeta ,\mu ,\nu$, and θ.

## 3. Quantum Discord in the WLQCC State

Quantum discord (QD) is a kind of quantum correlation quantifier proposed in 2001 [18]. In terms of definition, it is easy to prove that QD is invariant under local unitary operations on host qubits. Using this property, according to Equation (4), one can get

(9) $$\begin{array}{c} Q\left(\varrho_{ab}\right)=Q\left(\varrho_{ab}^{k}\right), \end{array}$$

Hence, to calculate QD one can directly consider the kernel state $\varrho_{ab}^{k}$ instead of the state $\varrho_{ab}$.

QD is defined as the discrepancy between two kinds of mutual information expressions which are identical in classical information theory. One kind of mutual information is generalized directly from classical mutual information. It is defined as $I\left(\rho_{ab}\right)=S\left(\rho_{a}\right)+S\left(\rho_{b}\right)-S\left(\rho_{ab}\right)$, where S(·) denotes the von Neumann entropy and $\rho_{a(b)}$ is a marginal state of $\rho_{ab}$. This kind of mutual information is called quantum mutual information, which is utilized to characterize and quantify the total correlation in state $\rho_{ab}$. For a WLQCC state $\varrho_{ab}$ in Equation (7), its total correlation can be obtained as

(10) $$\begin{array}{ccc} I(\varrho_{ab}^{k}) & = & S\left(\varrho_{a}^{k}\right)+S\left(\varrho_{b}^{k}\right)-S\left(\varrho_{ab}^{k}\right)\\ & = & 2-[\left(1-\sqrt{l_{a}}\right)\log_{2}\left(1-\sqrt{l_{a}}\right)+\left(1+\sqrt{l_{a}}\right)\log_{2}\left(1+\sqrt{l_{a}}\right)\\ & & +\left(1-\sqrt{l_{b}}\right)\log_{2}\left(1-\sqrt{l_{b}}\right)+\left(1+\sqrt{l_{b}}\right)\log_{2}\left(1+\sqrt{l_{b}}\right)]/2\\ & & +\log_{2}\Omega -\frac{1}{\Omega }\sum_{n=1}^{4}r_{n}\log_{2}r_{n}, \end{array}$$

where

(11) $$\begin{array}{c} l_{a(b)}=1-\left[{\left(4F-1\right)}^{2}\alpha_{a(b)}^{2}+2\left(1-F\right)\left(2F+1\right){\left(1+\alpha_{a(b)}^{2}\right)}^{2}\right]\frac{4\alpha_{b(a)}^{2}}{\Omega^{2}}, \end{array}$$

(12) $$\begin{array}{c} \left\{\begin{array}{c} r_{1}=\left[\Omega -2s_{1}-\sqrt{4s_{2}-\left(\Omega^{3}-4c_{1}\Omega +8c_{2}\right)/s_{1}}\right]/4,\\ r_{2}=\left[\Omega +2s_{1}-\sqrt{4s_{2}+\left(\Omega^{3}-4c_{1}\Omega +8c_{2}\right)/s_{1}}\right]/4,\\ r_{3}=\Omega /2-s_{1}-r_{1},\\ r_{4}=\Omega -r_{1}-r_{2}-r_{3}, \end{array}\right. \end{array}$$

and

(13) $$\begin{array}{c} \left\{\begin{array}{ccc} s_{1} & = & \sqrt{\Omega^{2}/4+\left\{s_{4}/s_{3}+s_{3}-2c_{1}\right\}/3},\\ s_{2} & = & \Omega^{2}/2-\left\{s_{4}/s_{3}+s_{3}+4c_{1}\right\}/3,\\ s_{3} & = & {\left[(s_{5}+\sqrt{s_{5}^{2}-4s_{4}^{3}})/2\right]}^{\frac{1}{3}},\\ s_{4} & = & c_{1}^{2}+12c_{3}-3c_{2}\Omega ,\\ s_{5} & = & 27\left(c_{2}^{2}+\Omega^{2}c_{3}\right)+c_{1}\left(2c_{1}^{2}-72c_{3}-9c_{2}\Omega \right), \end{array}\right. \end{array}$$

(14) $$\begin{array}{c} \left\{\begin{array}{ccc} c_{1} & = & 2\left(1-F\right)\left(2F+1\right)[\left(\alpha_{a}^{2}+\alpha_{b}^{2}\right)\left(\alpha_{a}^{2}\alpha_{b}^{2}+1\right)+2\alpha_{a}^{2}\alpha_{b}^{2}],\\ c_{2} & = & 4{\left(1-F\right)}^{2}\alpha_{a}^{2}\alpha_{b}^{2}\{6F\left(1+\alpha_{a}^{2}\right)\left(1+\alpha_{b}^{2}\right)\\ & & -\left(4F-1\right)[\left(1+\alpha_{a}^{2}\alpha_{b}^{2}\right)\left(1-\zeta \right)+\left(\alpha_{a}^{2}+\alpha_{b}^{2}\right)\zeta ]\},\\ c_{3} & = & 48F{\left(1-F\right)}^{3}\alpha_{a}^{4}\alpha_{b}^{4}. \end{array}\right. \end{array}$$

Actually, $r_{k}$s are the solutions of the quartic equation $r^{4}-\Omega r^{3}+c_{1}r^{2}-c_{2}r+c_{3}=0$ with *r* unknown. Three special cases should be clarified here: (1) when F=1, $r_{1}=\Omega$ and $r_{2}=r_{3}=r_{4}=0$; (2) when $\alpha_{a}=0$ and $\alpha_{b}=0$,$r_{1}=\Omega$ and $r_{2}=r_{3}=r_{4}=0$; (3) when $\alpha_{a}=1$ and $\alpha_{b}=1$, Equation (11) is not suitable anymore, and this case will be discussed in the analyses part of this paper. Easily, one can see that $I(\varrho_{ab})$ is a function of four parameters $F,\alpha_{a},\alpha_{b}$ and ζ.

Another kind of the mutual information is generalized from the classical analogy which is related to conditional entropy. It is defined as the maximal classical correlation $C_{a}\left(\rho_{ab}\right)=S\left(\rho_{b}\right)-\min_{\left\{{\hat{E}}_{a}^{i}\right\}}\sum_{i}p_{a}^{i}S\left(\rho_{b|i}\right)$ by optimizing over all possible measurements on part *a* (or *b*). Here $\rho_{b|i}={\mathrm{Tr}}_{a}\left({\hat{E}}_{a}^{i}⊗I_{b}\rho_{ab}\right)/{\mathrm{Tr}}_{ab}\left({\hat{E}}_{a}^{i}⊗I_{b}\rho_{ab}\right)$ is the state of part *b* conditioned on outcome *i* in part *a* with probability $p_{a}^{i}={\mathrm{Tr}}_{ab}\left({\hat{E}}_{a}^{i}⊗I_{b}\rho_{ab}\right)$, where $\left\{{\hat{E}}^{i}\right\}$ represents the set of positive-operator-valued measure (POVM) elements such that $\sum_{i}{\hat{E}}^{i}=I$. To get access to the maximal classical correlation, one must consider all possible POVM for optimization. As a matter of fact, the optimization is quite difficult for a generic state. In this study, von Neumann measurements are employed as an approximation. The measure element performed on subsystem *a* (or *b*) of the state $\rho_{ab}$ can be generally expressed as ${\hat{E}}^{i}=\hat{\Gamma }{\hat{P}}^{i}{\hat{\Gamma }}^{†},\;\;i=0,1,$ where ${\hat{P}}^{i}=\left|i\rangle \langle i\right|$ is the projector for the subsystem *a* (or *b*) along the computational bases |i〉 and $\hat{\Gamma }\in SU\left(2\right)$ is a unitary operator. $\hat{\Gamma }=\sqrt{\tau }e^{i\alpha }\left|0\rangle \langle 0\right|+\sqrt{1-\tau }e^{i\beta }\left|0\rangle \langle 1\right|-\sqrt{1-\tau }e^{-i\beta }\left|1\rangle \langle 0\right|+\sqrt{\tau }e^{-i\alpha }\left|1\rangle \langle 1\right|$ with parameters τ in the range 0≤τ≤1 and 0≤α,β≤π. Note that classical correlation is generally not a symmetric quantity, i.e., $C_{a}\left(\rho_{ab}\right)\neq C_{b}\left(\rho_{ab}\right)$. $C_{a}\left(\rho_{ab}\right)$ is usually referred to as the “left” classical correlation, while $C_{b}\left(\rho_{ab}\right)$ the ’right’ classical correlation.

For our concerned state $\varrho_{ab}^{k}$, it’s “left” classical correlation is a function of nine parameters $F,\alpha_{a},\alpha_{b},\zeta ,\mu ,\nu ,\tau ,\alpha$, and β before optimizing processes:

(15) $$\begin{array}{ccc} C_{a}\left(\varrho_{ab}^{k}\right) & = & S\left(\varrho_{b}^{k}\right)-\min_{\left\{{\hat{E}}_{a}^{i}\right\}}\sum_{i=0}^{1}p_{a}^{i}S\left(\varrho_{b|i}^{k}\right)\\ & = & 1-\frac{1}{2}\left(1-\sqrt{l_{a}}\right)\log_{2}\left(1-\sqrt{l_{a}}\right)-\frac{1}{2}\left(1+\sqrt{l_{a}}\right)\log_{2}\left(1+\sqrt{l_{a}}\right)\\ & & -\min_{\left\{\tau ,\alpha ,\beta \right\}}\sum_{i=0}^{1}p_{a}^{i}[1-\frac{1}{2}\left(1-\sqrt{1-\kappa_{a}^{i}}\right)\log_{2}\left(1-\sqrt{1-\kappa_{a}^{i}}\right)\\ & & -\frac{1}{2}\left(1+\sqrt{1-\kappa_{a}^{i}}\right)\log_{2}\left(1+\sqrt{1-\kappa_{a}^{i}}\right)]. \end{array}$$

where

(16) $$\begin{array}{c} \left\{\begin{array}{ccc} p_{a}^{0} & = & \{3\left[\alpha_{b}^{2}\tau +\alpha_{a}^{2}\left(1-\tau \right)\right]+\left(1-\alpha_{b}^{2}\right)\left[\tau \left(1+\alpha_{a}^{2}\right)-\alpha_{a}^{2}\right]\left[2\left(1-F\right)+\left(4F-1\right)\left(1-\zeta \right)\right]\\ & & -2\alpha_{a}\left(4F-1\right)\left(1-\alpha_{b}^{2}\right)\cos\left(\mu +\nu -\alpha -\beta \right)\sqrt{\zeta \tau (1-\zeta )(1-\tau )}\}/\Omega ,\\ p_{a}^{1} & = & 1-p_{a}^{0},\\ \kappa_{a}^{0} & = & 8\alpha_{b}^{2}\left(1-F\right)\left(2F+1\right){\left[\alpha_{a}^{2}+\tau \left(1-\alpha_{a}^{2}\right)\right]}^{2}/{\left(p_{a}^{0}\Omega \right)}^{2},\\ \kappa_{a}^{1} & = & 8\alpha_{b}^{2}\left(1-F\right)\left(2F+1\right){\left[1-\tau \left(1-\alpha_{a}^{2}\right)\right]}^{2}/{\left(p_{a}^{1}\Omega \right)}^{2}. \end{array}\right. \end{array}$$

To get the value of “left” classical correlation for a given Werner derivative ($F,\alpha_{a},\alpha_{b},\zeta ,\mu ,\nu$, and θ are given), one has to work out the extreme points of $\sum_{i=0}^{1}p_{a}^{i}S\left(\varrho_{b|i}\right)$ at first by solving the following three partial derivative equations:

(17) $$\begin{array}{c} \frac{\partial \sum_{i=0}^{1}p_{a}^{i}S\left(\varrho_{b|i}\right)}{\partial \tau }=0,\;\;\;\frac{\partial \sum_{i=0}^{1}p_{a}^{i}S\left(\varrho_{b|i}\right)}{\partial \alpha }=0,\;\;\;\frac{\partial \sum_{i=0}^{1}p_{a}^{i}S\left(\varrho_{b|i}\right)}{\partial \beta }=0. \end{array}$$

After inspecting the form of $\sum_{i=0}^{1}p_{a}^{i}S\left(\varrho_{b|i}\right)$, we find that parameters α and β induced by measurement are only present in $p_{a}^{0}$ in the form of cos(μ+ν−α−β). To simplify partial derivative procedures, we regard the function cos(μ+ν−α−β) as a new parameter *X* in the scope of [−1,1]. Thus the latter two equations in Equation (14) are transformed into the following two judgments:

(18) $$\begin{array}{c} \frac{\partial \sum_{i=0}^{1}p_{a}^{i}S\left(\varrho_{b|i}\right)}{\partial X}=0\;\;\mathrm{or}\;\;\frac{\partial X}{\partial \alpha }=\frac{\partial X}{\partial \beta }=\sin\left(\mu +\nu -\alpha -\beta \right)=0. \end{array}$$

Actually, it is still quite difficult to directly solve them. Fortunately, we find that the partial derivative equations hold if τ=0.5 and X=0. This implies that the point (τ,X)=(0.5,0) is an extreme point. Compare values of this extreme point with other special points, one easily finds that the former value is smaller than the latter ones. This means that this extreme point is at least a local minimal point. We conjecture that it is actually the global minimal point. To verify this conjecture, we have further investigated this feature via numerical calculations and conclusively confirmed that it is exactly the global minimal point. This global minimal point means that to achieve the “left” classical correlation for each state $\varrho_{ab}$, the optimal set of measurement $\left\{{\hat{E}}_{a}^{i}\right\}$ is determined by parameters τ=0.5 and α,β, where α,β are related to μ,ν by the equation cos(μ+ν−α−β)=0.

After substituting τ=0.5 and X=0 into Equation (12), we achieve the analytic expression for “left" classical correlation in state $\varrho_{ab}$:

(19) $$\begin{array}{ccc} C_{a}\left(\varrho_{ab}^{k}\right) & = & -\frac{1}{2}\left(1-\sqrt{l_{a}}\right)\log_{2}\left(1-\sqrt{l_{a}}\right)-\frac{1}{2}\left(1+\sqrt{l_{a}}\right)\log_{2}\left(1+\sqrt{l_{a}}\right)\\ & & +\frac{1}{2}\left(1-\Delta_{a}\right)\log_{2}\left(1-\Delta_{a}\right)+\frac{1}{2}\left(1+\Delta_{a}\right)\log_{2}\left(1+\Delta_{a}\right), \end{array}$$

where $\Delta_{a}=\sqrt{l_{a}+4\alpha_{a}^{2}\alpha_{b}^{2}{\left(4F-1\right)}^{2}/\Omega^{2}}$.

Now let us turn to the “right” classical correlation of $\varrho_{ab}$, it is a function of $F,\alpha_{a},\alpha_{b},\zeta ,\mu ,\nu$, and θ after optimized on parameters τ,α, and β which induced by the set of measurement elements $\left\{{\hat{E}}_{b}^{i}\right\}$:

(20) $$\begin{array}{ccc} C_{b}\left(\varrho_{ab}^{k}\right) & = & S\left(\varrho_{a}^{k}\right)-\min_{\left\{{\hat{E}}_{b}^{i}\right\}}\sum_{i=0}^{1}p_{b}^{i}S\left(\varrho_{a|i}^{k}\right)\\ & = & 1-\frac{1}{2}\left(1-\sqrt{l_{b}}\right)\log_{2}\left(1-\sqrt{l_{b}}\right)-\frac{1}{2}\left(1+\sqrt{l_{b}}\right)\log_{2}\left(1+\sqrt{l_{b}}\right)\\ & & -\min_{\left\{\tau ,\alpha ,\beta \right\}}\sum_{i=0}^{1}p_{b}^{i}[1-\frac{1}{2}\left(1-\sqrt{1-\kappa_{b}^{i}}\right)\log_{2}\left(1-\sqrt{1-\kappa_{b}^{i}}\right)\\ & & -\frac{1}{2}\left(1+\sqrt{1-\kappa_{b}^{i}}\right)\log_{2}\left(1+\sqrt{1-\kappa_{b}^{i}}\right)], \end{array}$$

where

(21) $$\begin{array}{c} \left\{\begin{array}{ccc} p_{b}^{0} & = & \{3\left[\alpha_{a}^{2}\tau +\alpha_{b}^{2}\left(1-\tau \right)\right]+\left(1-\alpha_{a}^{2}\right)\left[\tau \left(1+\alpha_{b}^{2}\right)-\alpha_{b}^{2}\right]\left[2\left(1-F\right)+\left(4F-1\right)\left(1-\zeta \right)\right]\\ & & +2\alpha_{b}\left(4F-1\right)\left(1-\alpha_{a}^{2}\right)\cos\left(\mu -\nu +\alpha +\beta +\theta \right)\sqrt{\zeta \tau (1-\zeta )(1-\tau )}\}/\Omega ,\\ p_{b}^{1} & = & 1-p_{b}^{0},\\ \kappa_{b}^{0} & = & 8\alpha_{a}^{2}\left(1-F\right)\left(2F+1\right){\left[\alpha_{b}^{2}+\tau \left(1-\alpha_{b}^{2}\right)\right]}^{2}/{\left(p_{b}^{0}\Omega \right)}^{2},\\ \kappa_{b}^{1} & = & 8\alpha_{a}^{2}\left(1-F\right)\left(2F+1\right){\left[1-\tau \left(1-\alpha_{b}^{2}\right)\right]}^{2}/{\left(p_{b}^{1}\Omega \right)}^{2}. \end{array}\right. \end{array}$$

To get the value of “right” classical correlation for a given Werner derivative, one has to work out the extreme points at first by solving the following three partial derivative equations:

(22) $$\begin{array}{c} \frac{\partial \sum_{i=0}^{1}p_{b}^{i}S\left(\varrho_{a|i}^{k}\right)}{\partial \tau }=0,\;\;\;\frac{\partial \sum_{i=0}^{1}p_{b}^{i}S\left(\varrho_{a|i}^{k}\right)}{\partial \alpha }=0,\;\;\;\frac{\partial \sum_{i=0}^{1}p_{b}^{i}S\left(\varrho_{a|i}^{k}\right)}{\partial \beta }=0 \end{array}$$

Similarly to the solution employed for the optimal procedure in dealing with “left" classical correlation, we regard the function cos(μ−ν+α+β+θ) as a new parameter *Y* in the scope of [−1,1]. Thus the latter two equations in Equation (19) are transformed into the following two judgments:

(23) $$\begin{array}{c} \frac{\partial \sum_{i=0}^{1}p_{b}^{i}S\left(\varrho_{a|i}^{k}\right)}{\partial Y}=0\;\;\mathrm{or}\;\;\frac{\partial Y}{\partial \alpha }=\frac{\partial Y}{\partial \beta }=-\sin\left(\mu -\nu +\alpha +\beta +\theta \right)=0. \end{array}$$

With the assistance of numerical calculations, we confirm that the point (τ,Y)=(0.5,0) is the optimal point for function $\sum_{i=0}^{1}p_{b}^{i}S\left(\varrho_{a|i}^{k}\right)$ for every state $\varrho_{ab}^{k}$. The optimal set of measurement elements $\left\{{\hat{E}}_{b}^{i}\right\}$ is determined by τ,α, and β, where τ=0.5 and α,β are determined by the values of μ,ν, and θ.

After substituting values of τ=0.5 and Y=0 into Equation (17), we achieve the analytic expression for “right” classical correlation in state $\varrho_{ab}^{k}$:

(24) $$\begin{array}{ccc} C_{b}\left(\varrho_{ab}^{k}\right) & = & -\frac{1}{2}\left(1-\sqrt{l_{b}}\right)\log_{2}\left(1-\sqrt{l_{b}}\right)-\frac{1}{2}\left(1+\sqrt{l_{b}}\right)\log_{2}\left(1+\sqrt{l_{b}}\right)\\ & & +\frac{1}{2}\left(1-\Delta_{b}\right)\log_{2}\left(1-\Delta_{b}\right)+\frac{1}{2}\left(1+\Delta_{b}\right)\log_{2}\left(1+\Delta_{b}\right), \end{array}$$

where $\Delta_{b}=\sqrt{l_{b}+4\alpha_{a}^{2}\alpha_{b}^{2}{\left(4F-1\right)}^{2}/\Omega^{2}}$.

The quantum discord of $\varrho_{ab}^{k}$ is defined as the discrepancy between the total correlation $I(\varrho_{ab}^{k})$ and the classical correlation $C_{a(b)}\left(\varrho_{ab}^{k}\right)$, i.e., $D_{a(b)}\left(\varrho_{ab}^{k}\right)=I\left(\varrho_{ab}^{k}\right)-C_{a(b)}\left(\varrho_{ab}^{k}\right)$. With the analytic expression of “left” and “right” classical correlations, we can achieve the analytical expression for “left” and “right” QD in state $\varrho_{ab}$:

(25) $$\begin{array}{ccc} Q_{a(b)}\left(\varrho_{ab}^{k}\right) & = & -\frac{1}{2}\left(1-\sqrt{l_{a(b)}}\right)\log_{2}\left(1-\sqrt{l_{a(b)}}\right)-\frac{1}{2}\left(1+\sqrt{l_{a(b)}}\right)\log_{2}\left(1+\sqrt{l_{a(b)}}\right)\\ & & -\frac{1}{2}\left(1-\Delta_{a(b)}\right)\log_{2}\left(1-\Delta_{a(b)}\right)-\frac{1}{2}\left(1+\Delta_{a(b)}\right)\log_{2}\left(1+\Delta_{a(b)}\right)\\ & & +2-\log_{2}\Omega +\frac{1}{\Omega }\sum_{k=1}^{4}r_{k}\log_{2}r_{k}. \end{array}$$

## 4. Analyses and Discussions

In the previous section, we have obtained the analytic expressions of the “left” and “right” QDs in the WLQCC states. According to the analytical expression in Equation (25), in this section, we will make some analyses and discussions on these obtained QDs.

Firstly we want to emphasize that, we have developed and used some skills to obtain the analytic expressions of the QD, as is a distinct feature of our present study. In this paper, the concerned state is the WLQCC state $\varrho_{ab}$ (see in Equation (2)). Due to the complicated form of LQCC protocol, the number of parameters needed to define the state $\varrho_{ab}$ is great. The corresponding number of independent parameters is 19. Hence, it is very difficult to consider the state $\varrho_{ab}$ directly. Fortunately, after some analyses, we find one can use some skills to degrade this difficulty. To be concrete, noticing that our main purpose in this paper is to calculate the QD in the WLQCC state $\varrho_{ab}$. Moreover, $\varrho_{ab}$ is related to $\varrho_{ab}^{k}$ (see in Equation (5)) with local unitary transformations and QD is invariant under the transformations. Hence, in calculating QD in $\varrho_{ab}$ one can consider the kernel state $\varrho_{ab}^{k}$ instead. The parameters needed in $\varrho_{ab}^{k}$ is 7. Hence, the quantity and complexity in calculating QD are reduced to a great extent. See Table 1.

Furthermore, through complicated calculation and derivation, one can find that the final number of the independent parameters defining QD in the concerned state $\varrho_{ab}^{k}$ is 4. That is to say, the final analytical expression obtained from the QD of the WLQCC states is only a function of 4 independent parameters, i.e., *F*, $\alpha_{a}$, $\alpha_{b}$, and ζ. Using the above skills and derivations, the difficulty is evidently degraded and the analytic expression is obtained.

**Figure 1:**
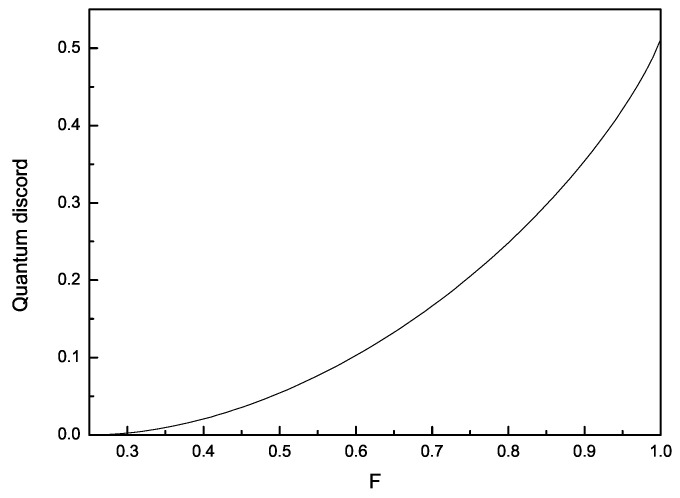
The variation of quantum discord $Q_{a}$ with *F* for $\alpha_{a}$ = 0.3, $\alpha_{b}$ = 0.6, and ζ = 0.8.

**Figure 2:**
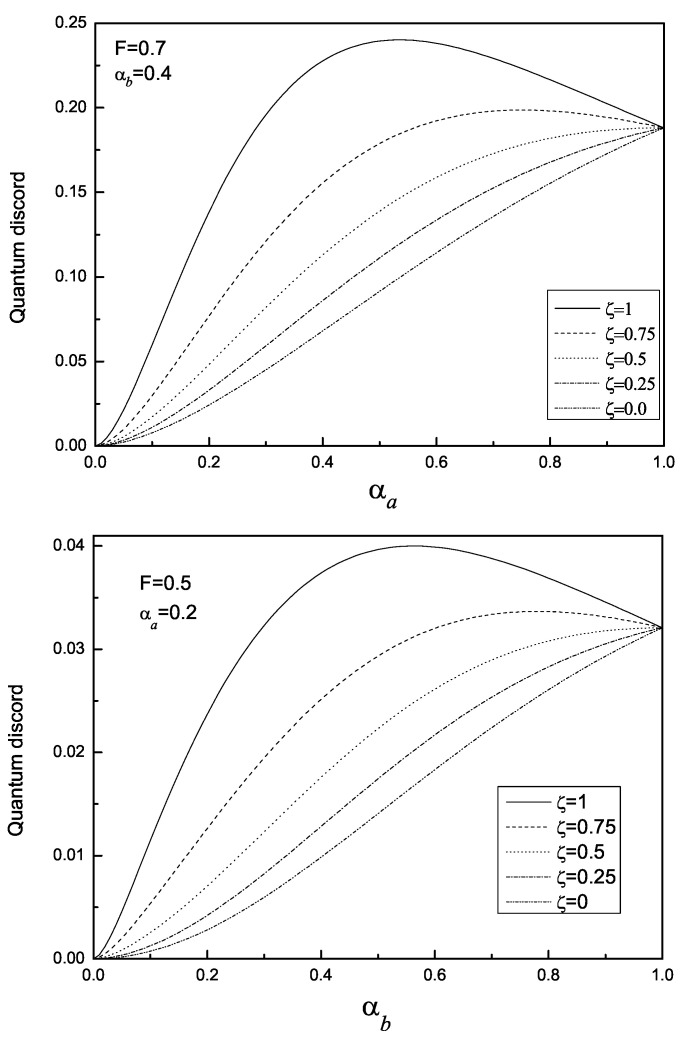
The variation of quantum discord $Q_{a}$ with $\alpha_{a}$ or $\alpha_{b}$.

**Figure 3:**
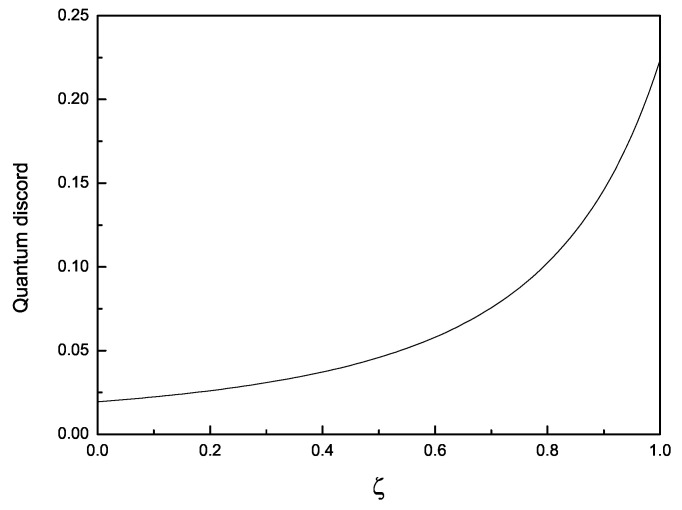
The variation of quantum discord $D_{a}$ with ζ for given F = 0.8, $\alpha_{a}$ = 0.2, $\alpha_{b}$ = 0.3.

The second, let us move to investigate the features in the obtained QD. According to the symmetry of the ’left’ quantum discord and the “right” one, in the following, we will make some analyses about the variation of the “left” quantum discord $D_{a}$ with *F*, $\alpha_{a}$, $\alpha_{b}$, and ζ. Through analyses, one can get the following distinct features.

(i) $D_{a}$ is an increasing function of *F* for any given set of $\left(\alpha_{a},\alpha_{b},\zeta \right)$ and reaches its maxima at F=1 (See Figure 1). This phenomenon can be understood as follows. Inspect the original Werner state in Equation (1) and the WLQCC state in Equation (2). Originally, state $\rho_{ab}^{W}$ consists of two terms, which are mixed with the weights characterized by *F*. The former represented by *I* is the maximally mixed state with no quantum correlation. The latter is the maximally entangled state. In this case, a bigger *F* corresponds to a larger weight of the latter term and accordingly a larger quantum correlation is induced by the mixture of the two terms. As for the WLQCC state $\varrho_{ab}$ which are mapped from the original state $\rho_{ab}^{W}$, quantum correlation in it still increases with the parameter *F*. This means that the WLQCC operation do not change the dependency relation about QC with *F*. However, according to [40], one can find that for a given *F* the quantity of QC in $\varrho_{ab}$ is less than that in $\rho_{ab}^{W}$. This means that the nonzero set of $\left(\alpha_{a},\alpha_{b},\zeta \right)$ can decrease the quantum discord for a given *F*. That is to say, the LQCC protocol cannot increase the quantum discord in the Werner state $\rho_{ab}^{W}$.

(ii) For given ζ∈[0,0.5], *F*, and $\alpha_{b}$, $D_{a}$ monotonically increases with $\alpha_{a}$ and reaches its maxima at $\alpha_{a}=1$ (See Figure 2). However, for given ζ∈[0.5,1.0], *F*, and $\alpha_{b}$, $D_{a}$ first increases then decreases with $\alpha_{a}$, and the transition point is determined by the exact value of ζ. As for the case of $\alpha_{b}$, one can find that the dependent relation of $D_{a}$ on $\alpha_{b}$ for given ζ, *F* and $\alpha_{a}$ is similar to that on $\alpha_{a}$. This feature is related to the LQCC operation and its underlying physics can be explained as follows. The LQCC operation is concerned with two parties, Alice and Bob, who each control one subsystem, and who are restricted to carrying out local quantum operations and classical communication (i.e., LQCC). Specifically, the quantum operations Alice and Bob are allowed to perform are local unitary transformations and local filtrations. As analyzed in the second section in this paper, to calculate the quantum discord in the WLQCC state $\varrho_{ab}$ in Equation (2), we have considered the kernel state $\varrho_{ab}^{k}$ in Equation (5) instead. From Equation (5), one can see that $\alpha_{a}$ is the parameter characterizing the filtration operator $f_{a}^{\prime }$, $\alpha_{b}$ is the one for $f_{b}^{\prime }$, and ζ is the one for the unitary operator $W_{a}$.

(iii) Through analysis, one can find that quantum discord $D_{a}$ is a monotonically increasing function of ζ for given $\alpha_{a}$, $\alpha_{b}$, and *F*. See Figure 3 for example. ζ is the parameter used to characterize the unitary operation $W_{a}$. In addition, according to the expression of quantum discords in WLQCC states, the parameter ζ vanishes when $\alpha_{a}=1$ or $\alpha_{b}=1$. Especially, in the situation of $\alpha_{a}=\alpha_{b}=1$, LQCC is turned into local unitary operation, which does not change the quantum discords in Werner states. When $\alpha_{a}=0$ or $\alpha_{b}=0$, the total correlations of WLQCC states are zero. The special case $\alpha_{a}=\alpha_{b}=0$ is meaningless for the matrix form of WLQCC states is zero.

Finally, we want to stress that, although the result, i.e., LQCC cannot increase the QD (similar to the behavior of quantum entanglement), has been obtained, it is still an open question whether this conclusion is applicable for other states or in the framework of other QCDEs. In fact, it is easy to verify that this conclusion is not general. In other words, for other general states and for other quantum correlation measures, the property of LQCC cannot increase and the quantum correlation may not exist at all. Nonetheless, for other states with high inherent property of symmetry, such as the higher-dimensional (qutrit) Werner states, we conjecture that the coincidence may remain. We will pay attention to it in the near future.

## 5. Summary

To summarize, in this paper, we have derived the analytical expression of QDs in WLOCC states. With the assistance of numerical computations, we find that the QD in a WLQCC state cannot exceed that of the original Werner state. The research in [PRL 81, 3279] exhibits a similar result in the scenario of quantum entanglement: QDs in two-qubit Werner states cannot be increased by single-state LQCC protocols.

## Figures and Tables

**Table 1:** The number of parameters of Werner states by local quantum operations and classical communication protocol (WLQCC) states, kernel states, and their quantum discord. N denotes the number of parameters.

	$\varrho_{\mathrm{ab}}$ (Equation (2))	$\varrho_{\mathrm{ab}}^{k}$ (Equation (7))	$Q\left(\varrho_{\mathrm{ab}}\right)=Q\left(\varrho_{\mathrm{ab}}^{k}\right)$
*N*	19	7	8
